# The Kynurenine Pathway and Mediating Role of Stress in Addictive Disorders: A Focus on Alcohol Use Disorder and Internet Gaming Disorder

**DOI:** 10.3389/fphar.2022.865576

**Published:** 2022-04-11

**Authors:** Joon Hwan Jang, So Young Yoo, Yae Eun Park, Mi-Jung Ji, Hyun-Mee Park, Ji Hyun Back, Ji Yoon Lee, Dai Jin Kim, Ji Eun Lee, Jung-Seok Choi

**Affiliations:** ^1^ Department of Psychiatry, Seoul National University Health Service Center, Seoul, South Korea; ^2^ Department of Human Systems Medicine, Seoul National University College of Medicine, Seoul, South Korea; ^3^ Department of Psychiatry, SMG-SNU Boramae Medical Center, Seoul, South Korea; ^4^ Center for Theragnosis, Biomedical Research Division, Korea Institute of Science and Technology, Seoul, South Korea; ^5^ Advanced Analysis Center, Research Resources Division, Korea Institute of Science and Technology, Seoul, South Korea; ^6^ Department of Biotechnology, College of Life Sciences and Biotechnology, Korea University, Seoul, South Korea; ^7^ Department of Psychiatry, Samsung Medical Center, Seoul, South Korea; ^8^ Department of Psychiatry, Seoul St. Mary’s Hospital, The Catholic University of Korea College of Medicine, Seoul, South Korea

**Keywords:** stress, kynurenine pathway, executive function, addiction, alcohol use disorder, internet gaming disorder

## Abstract

Stress plays an important role in the pathophysiology of addictive disorders. The kynurenine (KYN) pathway involved in neuroimmune and cognitive functions is activated under stress. However, the neuroimmunological–neurocognitive mechanisms in the role of stress in addictive disorders are unclear still now. Ninety-nine young adults aged 18–35 years [alcohol use disorder (AUD), *N* = 30; Internet gaming disorder (IGD), *N* = 34; healthy controls (HCs), N = 35] participated in this study. Stress levels, resilience, addiction severity, and neurocognitive functions were evaluated, and serum levels of tryptophan (TRP), 5-hydroxytryptamine (5-HT), KYN, and kynurenine acid (KYNA) were determined using liquid chromatography coupled with tandem mass spectrometry through blood samples. Both addictive disorder groups showed higher levels of stress, lower resilience, and impaired executive functions compared to the HC group. Importantly, the AUD group revealed significantly increased KYN levels and KYN/TRP ratios, as well as decreased KYNA levels and KYNA/KYN ratios compared to HCs (*p* < 0.001, *p* < 0.001, *p* = 0.033, and *p* < 0.001, respectively). The IGD group showed KYN levels and KYNA/KYN ratios intermediate between those of the AUD group and HCs. Furthermore, in the AUD group, the mediating effect of AUD on KYN through stress level was moderated by resilience [index of moderated mediation = −0.557, boot S.E = 0.331, BCa CI (−1.349, −0.081)]. Stress may induce an imbalance in downstream of KYN pathway metabolites, and the KYN/TRP ratio may play as a neuromediator between stress and behavioral changes in both addictive disorders. This study suggests that regulation of the KYN pathway is critical in the pathophysiology of addictive disorders and it may serve as an important target for future treatment modalities.

## Introduction

Considerable evidence has emphasized the importance of stress in the development of addictive disorders (Hildebrandt and Greif, 2013; Koob and Schulkin, 2019; Ruisoto and Contador, 2019). Chronic stress is one of the major sources of allostatic load, bringing some brain changes and increasing the risk of addiction (Koob and Schulkin, 2019; Fronk et al., 2020). Various psychological and physical stressors can lead to long-term changes in the brain (Ruisoto and Contador, 2019; Wojdala et al., 2020). Repeated exposures to addictive stimuli and withdrawal from those stimuli can be stressors to the brain and potentially induce brain changes and possibilities of relapse (George et al., 2014). Therefore, stress has been known as the single most powerful and reliable trigger for cravings and relapse (Sinha, 2008; Wand, 2008; Preston et al., 2018). In contrast, resilience—the ability to be healthy and adaptable and to cope positively with changes (Southwick et al., 2014) is considered a protective factor against the development of addictive disorders (Nam et al., 2018; Shin et al., 2019; Ersche et al., 2020). Therefore, understanding the influence of stress and resilience on vulnerability to addiction is crucial in the context of effective prevention and treatment.

The kynurenine (KYN) pathway is one of tryptophan (TRP) metabolism pathways that are activated under stress and/or immune system activation. Under normal conditions, TRP is initially converted to KYN, which is subsequently converted to various metabolites with neuromodulatory properties (O'Farrell and Harkin, 2017). Pro-inflammatory cytokines are critical inducers of TRP-2,3-dioxygenase (TPO) and indoleamine-2,3-dioxygenase (IDO), which are rate-limiting enzymes in the KYN pathway (Zhou et al., 2019). The KYN/TRP ratio is a measure of IDO activity, indicating neurotoxicity (Zhou et al., 2019). Kynurenic acid (KYNA), which is produced from KYN by kynurenine aminotransferase (KAT), is often considered a neuroprotective KYN pathway metabolite due to its antagonistic properties at the N-methyl-d-aspartate (NMDA) receptor (Stone, 1993).

In addition, abnormalities in the KYN pathway are thought to contribute to the pathogenesis of cognitive deficits, as peripheral TRP exhaustion increases the rate of TRP degradation and the imbalance between the neurotoxic metabolite quinolinic acid (QUIN) and the neuroprotective metabolite KYNA via KYN monooxygenase (KMO) (Zhou et al., 2019). Previous studies showed that the activation of IDO plays a critical role in cognitive deficits (Heisler and O'Connor, 2015).

Research in patients with alcohol use disorder (AUD) showed that alcohol-related variables such as the severity of AUD or duration of abstinence were associated with changes in KYN levels and the KYN/TRP ratio (Neupane et al., 2015). Vidal et al. (2020) also reported that patients with AUD exhibited higher KYN concentrations and lower KYNA concentrations than those of the healthy controls. Lower plasma KYNA concentrations have been observed in abstinent patients with cocaine use disorder (Araos et al., 2019). These findings suggest that KYN pathway metabolites may be important biochemical components in substance use disorders.

The relationship between stress and the KYN pathway suggests that the pro-inflammatory status associated with stressful experiences in addictive disorders can activate the enzymes IDO and KMO, resulting in dysregulation of the KYN pathway. However, there have been no reports of the KYN pathway being involved in behavioral addictions such as Internet gaming disorder (IGD). Furthermore, it is still unclear how the neuroimmunological–neurocognitive mechanisms work in the effects of stress on addictive disorders.

In this study, we investigated the serum concentrations of KYN pathway metabolites—including TRP, 5-hydroxytryptamine (5-HT), KYN, and KYNA—as well as stress levels, resilience, and neurocognitive functions in young adults with AUD and IGD and healthy controls (HCs). Furthermore, we explored the associations between KYN pathway metabolites and stress, resilience, neurocognitive functions, and symptom severity in the addiction group. We hypothesized that patients with addictive disorders would show higher levels of stress, lower resilience, and impaired executive functions, as well as shifts to stress responses in the KYN pathway compared to the HC group. We further hypothesized that altered levels of KYN pathway metabolites would be associated with the level of stress, resilience, executive deficits, and symptom severity in the AUD group and IGD group.

## Materials and Methods

### Participants

Ninety-nine young adults were recruited from the SMG-SNU Boramae Medical Center and the surrounding community in Seoul, Republic of Korea. Their age was from 18 to 35 years. AUD and IGD were diagnosed by a clinically experienced psychiatrist based on the criteria of the Diagnostic and Statistical Manual of Mental Disorders, Fifth Edition (DSM-5). Patients with AUD or IGD had no comorbid psychiatric diagnoses, including attention deficit hyperactivity disorder, depressive or anxiety disorders, a history of head injury, clinically important medical illness, or cognitive impairments. All participants were drug-naive during the assessments.

Alcohol Use Disorder Identification Test (AUDIT) was used to estimate the severity of AUD. The mean amount of alcohol consumption in this group was 11.63 ± 6.57 standard drinks per day. Patients with AUD played Internet games for less than 2 h per day and had abstained from alcohol use for at least 2 weeks before participation in the study. Abstinence from alcohol was checked through self-reports and reports from caregivers.

The severity of IGD was evaluated via Young’s Internet Addiction Test (Y-IAT). The average time spent playing Internet games per weekday and weekend day were 6.45 ± 3.71 and 8.74 ± 3.40 h, respectively. Patients with IGD consumed fewer than 14 standard drinks per week. None of the participants met the criteria for both AUD and IGD.

The HC group was recruited from the local community. HCs spent less than 2 hours per day playing Internet games and drank fewer than 14 standard drinks per week. They had no lifetime history of any psychiatric disorders, including IGD and AUD.

Venous blood sampling was conducted before the examination of clinical and neurocognitive functioning. All participants underwent The Korean version of the Wechsler Adult Intelligence Scale-IV (WAIS-IV) to evaluate the intelligence quotient (IQ); only subjects with WAIS-IV scores >80 were included. These tests were completed in approximately 150 min. All participants were administered the tests on a single session. This study protocol was approved by the Institutional Review Board of SMG-SNU Boramae Medical Center and was conducted by the Declaration of Helsinki. All participants received detailed information about the study procedure and provided written informed consent.

### Clinical Measures

#### Young’s Internet Addiction Test

Y-IAT measured the severity of IGD Total scores were calculated according to Young’s method (Young, 1998; Beard and Wolf, 2001), with possible total scores ranging from 20 to 100. The Cronbach’s alpha coefficient was 0.96.

#### Alcohol Use Disorder Identification Test

The Korean version of the AUDIT (Kim et al., 1991) was used to assess the severity of AUD This scale asks the frequency of alcohol abuse behavior and contains 10 questions. Total scores range from 0 to 40, with higher scores indicating greater dependence on alcohol. The Cronbach’s alpha coefficient was 0.92.

#### Psychosocial Well-being Index

Stress levels were measured using the PWI, which contains 45 items (Kim, 1999). The PWI was developed based on the General Health Questionnaire generated by Goldberg (Goldberg and Hillier, 1979; Derose, 2004), which evaluates psychological stability among community populations and was modified to fit the context of the Korean population (Kim, 1999). The PWI contains questions addressing the respondents’ social roles, self-confidence, depression, sleep disturbances, anxiety, and general well-being. Total scores range from 0 to 135, with higher scores indicating greater distress. The Cronbach’s alpha coefficient was 0.91.

#### Connor–Davidson Resilience Scale

Resilience was assessed via the CD-RISC (Connor and Davidson, 2003) with a 25-items self-report instrument. The CD-RISC shows how a participant felt over the past month; total scores range from 0 to 100, with higher scores reflecting greater resilience. The Cronbach’s alpha coefficient was 0.96.

#### Beck Depression Inventory-II

The BDI is a 21-item self-reporting scale that measures the presence and severity of depressive symptoms during the past week (Beck et al., 1996; Lim et al., 2011). . The Cronbach’s alpha coefficient was 0.98.

#### Beck Anxiety Inventory

The BAI is a 21-question self-reporting inventory and uses a four-point scale to measure anxiety (Beck et al., 1988; Kwon, 1997). Scores for the 21 items are combined to yield a single anxiety score. The Cronbach’s alpha was 0.96.

### Executive Function

#### Color–Word Stroop Test

The Korean Color–Word Stroop Test (K-CWST) (Kim et al., 2004) was used for the measurement of interference control. In the color–word condition, participants are asked to name, as quickly as possible, the font color of a color word on the presented card, where the actual color word differs from the font color (e.g., the word “red” printed in blue font). Thus, participants must inhibit their automatic reading process during the K-CSWT.

#### Trail-Making Test

The TMT assesses motor planning (type A) and cognitive flexibility related to compulsivity (type B) (Reitan, 1992). The task asks participants to concentrate on a sequence of consecutive targets on a computer screen. The TMT-A asks participants to read numbers as quickly as possible and the result reflects visuospatial searchability. The TMT-B, which requires participants to read numbers and letters alternately, additionally measures the ability to perform cognitive shifts. The total times for completing parts A and B were used as the dependent variables (Tombaugh, 2004).

#### Spatial Working Memory and Spatial Span

In the present study, SWM and SSP were measured using the Cambridge Neuropsychological Test Automated Battery (CANTAB) (http://www.camcog.com).

SWM requires the retention and manipulation of visuospatial information. This self-ordered test requests notable executive function demands and measures strategies such as working memory errors. An efficient strategy is to follow a predetermined sequence beginning with a specific box and then, once a token has been found, returning to that box to start a new search sequence (Owen et al., 1990). The use of this strategy is estimated by counting the number of times a participant began a new search with a box different from the last search; thus, a high score represents an inefficient strategy.

SSP is a visuospatial version of the digit-span test to assess attention/working memory. The participants were asked to sequentially mimic flashing boxes following the order presented. Outcome measures are span length (the longest sequence successfully recalled) and a number of errors.

### Analysis of Kynurenine Pathway Metabolites

#### Chemicals and Reagents

Standard products (TRP, 5HT, KYN, and KYNA) and bovine serum albumin (BSA) were purchased from Sigma-Aldrich (St. Louis, MO, United States). The internal standards, TRP-^13^C_11_, 5-HT-d_4_, and KYNA-d_5_, were purchased from Cambridge Isotope Laboratories Inc. (Tewksbury, MA, United States), TLC Pharmaceutical Standards Ltd. (Aurora, Ontario, Canada), and Toronto Research Chemical Inc. (Toronto, Ontario, Canada), respectively. Methanol was obtained from Honeywell Burdick & Jackson (Ulsan, Korea), and dimethyl sulfoxide (DMSO) and phosphate buffered saline (PBS) were purchased from Samchun Pure Chemicals (Pyeongtaek, Korea). Acetonitrile (ACN), water, and formic acid (FA) were purchased from Merck (Billerica, MA, United States).

#### Preparation of Standard Solutions

Individual stock solutions of TRP, 5-HT, KYNA, TRP-^13^C_11_, and 5-HT-d_4_ were prepared at 1 mg/ml in 0.1% FA in 50% methanol. Stock solutions of KYNA and KYNA-d_5_ were prepared at a concentration of 1 mg/ml in DMSO. Then, the stock solutions of TRP, 5-HT, KYNA, and KYN were mixed and diluted to 20 μg/ml using 50% ACN in water and stored at −20°C until use.

#### Sample Preparation

Human serum samples (10 μL) were mixed with 30 μL of cold ACN containing an internal standard mixture (50 ng/ml of TRP-^13^C_11_, 5-HT-d_4_, and KYNA-d_5_) by vortexing for 5 min and incubating on ice for 10 min, followed by centrifugation at 13,000 × *g* at 4°C. The supernatant (30 μL) of each serum sample was then mixed with 30 μL of 0.1% FA in water.

#### Liquid Chromatography Coupled With Tandem Mass Spectrometry

TRP, 5-HT, KYN, and KYNA levels in serum samples were analyzed by LC-MS/MS. An Agilent 1290 Infinity II LC system (Agilent Technologies, Palo Alto, CA, United States) was interfaced with a QTRAP 6500 + system (AB SCIEX, Toronto, ON, Canada) equipped with an electrospray ionization (ESI) source. The analytes were separated on an Acquity UPLC HSS T3 column (2.1 × 100 mm, 1.8 μm, Waters, MA, United States) with an Acquity UPLC HSS T3 VanGuard column (2.1 × 5 mm, 1.8 μm, Waters, MA, United States). The column temperature was set at 40°C. The mobile phase consisting of 0.1% FA in water (buffer A) and ACN (buffer B) was delivered at a flow rate of 500 μL/min with the following gradient conditions: 0–1.5 min 100% buffer A (0.1% FA in water), 1.five to six min 0–60% B, 6–7.2 min 60–95% B, 7.2–7.5 min 95% B, 7.5–7.8 min 95–0% B. The injection volume of each sample was 5 μL. The mass spectrometer was operated at the following parameters: ion spray voltage, 5.5 kV; source temperature, 600°C; curtain gas, 35 psi; nebulizer gas (GS1), 50 psi; and heating gas (GS2), 50 psi. The MS/MS data were acquired in the time-scheduled multiple reaction ion monitoring (MRM) mode in the ESI-positive mode. The MRM monitoring conditions for each compound are summarized in Supplementary Table S1. Two ion transitions were selected for each analyte, one for quantitative analysis and the other for qualitative purposes, to confirm identity. Instrument control, data acquisition, and analysis were performed using Analyst 1.6.3 (AB SCIEX, Toronto, ON, Canada).

#### Calibration

The calibration samples were prepared by adding the mixed standard solutions (TRP, 5-HT, KYN, and KYNA) to 90 μL of cold ACN containing an internal standard mixture (50 ng/ml of TRP-^13^C_11_, 5-HT-d_4_, and KYNA-d_5_). Then, the samples were vortexed for 5 min and incubated on ice for 10 min, followed by centrifugation at 13,000 × *g* at 4°C. The supernatant (50 μL) of each calibration sample was then mixed with 50 μL of 0.1% FA in water, and 5 μL of the calibration sample was injected into the LC-MS/MS system. The ranges of the calibration curves are shown in Supplementary Table S2. The calibration curves were constructed by plotting the peak area ratios of all analytes versus their internal standards against the concentrations of the calibration standards. Linearity was evaluated using the correlation coefficient, which is the *R* ^*2*^ value of the calibration curve. The standard curve exhibited good linearity (R^2^ > 0.99) for all analytes.

**FIGURE 1:**
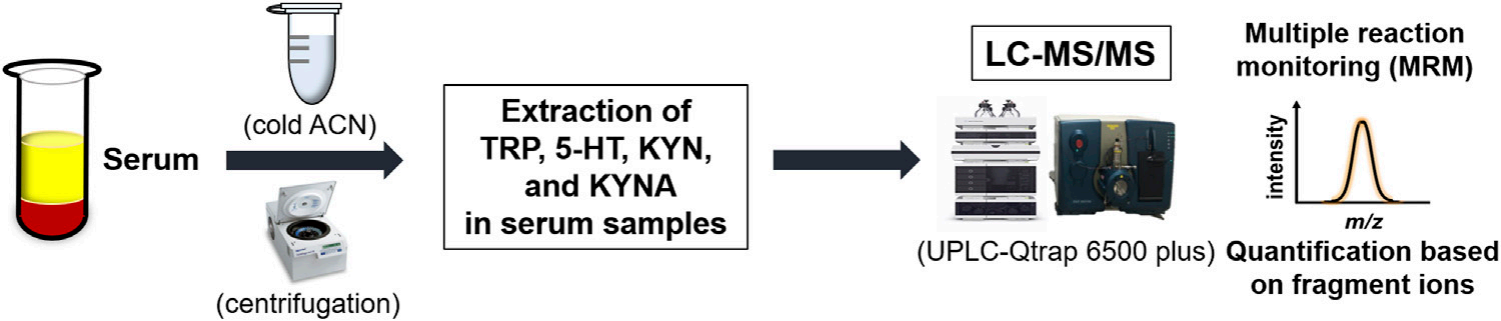
Workflow of analysis of kynurenine pathway metabolites in serum samples using liquid chromatography coupled with tandem mass spectrometry (LC-MS/MS). ACN, Acetonitrile; TRP, Tryptophan; 5-HT, 5-hydroxytryptamine; KYN, Kynurenine; KYNA, Kynurenic acid.

The workflow of the analysis of KYN pathway metabolites in the present study is summarized in Figure 1.

### Statistical Analyses

**FIGURE 2:**
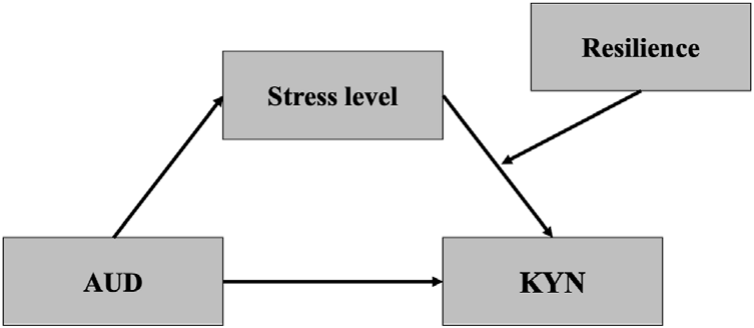
The moderated mediation model in the present study. AUD, Alcohol Use Disorder; KYN, Kynurenine.

We conducted the chi-squared test and one-way analysis of variance to compare demographic and clinical characteristics among the three groups. Regarding neurocognitive functions, the between-group analysis was adjusted for IQ, BDI, and BAI due to their associations with executive function variables. In addition, the between-group analysis of KYN pathway metabolites was adjusted for BDI and BAI due to their associations with KYN pathway metabolites. Bonferroni’s test was used for *post hoc* analysis. Pearson’s correlation coefficients were calculated to identify relationships between stress levels, resilience, and executive function with KYN metabolites and severity of the addiction as measured by AUDIT or Y-IAT in each patient group. In the case of variables exhibiting significant correlations, simple linear regression analyses were conducted as exploratory analyses to examine whether each variable could predict the level of KYN pathway metabolites or severity of addiction in the AUD or IGD group. Further, the indirect effects, conditional effects, and conditional indirect effects were analyzed using a bootstrapping approach, the PROCESS macro version 3.5 for SPSS (Hayes, 2017). Before the analysis, we divided the cohort into clinical groups and HCs (AUD and HCs; IGD and HCs) to evaluate model effects in each group. We conducted simple mediation models (Model 4) to figure out whether stress level mediated the relationship between AUD (AUD = 1; HCs = 0) or IGD (IGD = 1; HCs = 0) and KYN pathway metabolites. Second, simple moderation models (Model 1) were applied to confirm that stress level interacted with resilience and KYN pathway metabolites. Third, moderated mediation models (Model 14) were accessed to determine whether resilience moderates the indirect effect of IGD or AUD on KYN pathway metabolites through stress level (Figure 2). Significance was determined using bias-corrected 95% confidence intervals (BCa CI) by bootstrapping using 5,000 resamples. We used the Sobel test (Z) to examine the indirect effect size of the mediating models (Preacher and Leonardelli, 2001). Conditional effects at plus and minus one standard deviation around the mean of resilience were calculated using the pick-a-point method. Continuous variables were mean-centered to reduce any multicollinearity, and BDI, BAI, and IQ scores were controlled in all mediation analyses.

Statistical significance was set at *p* < 0.05. Statistical analysis was performed using SPSS 26.0 (IBM Corp., Armonk, NY, United States).

## Results

### Demographic, Clinical, and Neurocognitive Variables

Table 1 summarizes participants’ demographic, clinical, and neurocognitive information. There were no significant differences in age and sex among the groups, although the education level was significantly higher in the HC group than in the AUD and IGD groups.

**TABLE 1:** Demographic, clinical and neurocognitive variables in the alcohol use disorder, Internet gaming disorder and healthy control groups.

	Healthy control	Internet gaming disorder	Alcohol use disorder			
	*N* = 35	*N* = 34	*N* = 30	χ^2^, F	*p*	Post-hoc
	mean ± SD	mean ± SD	mean ± SD			
*Demographic domains*						
Age (years)	24.657 ± 2.990	23.559 ± 4.230	27.233 ± 5.117	1.942	0.149	
Sex (M/F)	32/3	31/3	22/8	5.563	0.062	
Education (years)	14.486 ± 1.821	13.382 ± 1.498	13.633 ± 1.564	4.281	0.017	I < H
*Clinical domains*						
AUDIT	4.743 ± 3.355	5.265 ± 6.593	26.933 ± 6.757	153.779	<0.001	A > I,H
Y-IAT	27.914 ± 6.528	62.588 ± 16.463	35.333 ± 13.555	69.272	<0.001	I > A,H
PWI	23.829 ± 14.329	55.882 ± 20.908	64.267 ± 25.216	36.642	<0.001	A,I > H
CD-RISC	74.286 ± 11.253	51.235 ± 18.627	49.000 ± 18.300	24.915	<0.001	A,I < H
BDI	2.257 ± 2.737	16.382 ± 10.070	18.467 ± 13.952	27.150	<0.001	A,I > H
BAI	2.229 ± 2.669	15.706 ± 13.588	16.800 ± 13.010	18.987	<0.001	A,I > H
*Neurocognitive domains*						
IQ	116.829 ± 10.397	100.853 ± 21.241	100.655 ± 11.657	12.335	<0.001	A,I < H
TMT-A (sec)	20.114 ± 7.749	26.529 ± 13.415	23.069 ± 6.606	2.436	0.093	
TMT-B (sec)	50.571 ± 14.555	87.471 ± 103.782	66.103 ± 32.256	4.216	0.018*	I > H
Stroop-word (sec)	51.943 ± 8.314	59.235 ± 11.471	56.517 ± 6.577	1.071	0.347	
Stroop-color (sec)	89.229 ± 14.330	104.353 ± 29.354	98.414 ± 11.163	2.467	0.090	
SWM-total	8.114 ± 10.471	15.176 ± 20.589	19.655 ± 15.655	0.618	0.541	
SWM-strategy	27.743 ± 4.865	28.971 ± 6.640	32.448 ± 5.040	3.317	0.041*	A > I,H
SSP-length	8.343 ± 0.968	7.618 ± 1.415	7.138 ± 1.329	1.722	0.184	
SSP-error	8.371 ± 7.232	11.324 ± 6.861	11.483 ± 5.604	0.143	0.867	

SD: standard deviation; M: male; F:female; AUDIT: alcohol use disorder identification test; Y-IAT: Young’s Internet Addiction Test; PWI: Psychosocial Well-being Index; CD-RISC: Connor-Davidson Resilience Scale; BDI: beck depression inventory; BAI: beck anxiety inventory; IQ: intelligence quotient; TMT: trail making test; SWM: spatial working memory; SSP: spatial span; p-value in the analysis of neurocognitive domains was adjusted by IQ, BDI, and BAI. the bonferroni test was used for post hoc analyses.

*p < 0.05.

Clinical differences were significantly shown among the groups. Both the AUD and IGD groups had significantly higher mean scores of PWI, BDI, and BAI than the HCs (*p* < 0.001), indicating that the AUD group or IGD group had increased levels of stress and symptoms of depression and anxiety. Furthermore, both the AUD and IGD groups had significantly lower mean CD-RISC scores than the HCs (*p* < 0.001), indicating that both patient groups exhibited reduced levels of resilience. The AUD group had higher AUDIT scores than the IGD and HC groups (*p* < 0.001), and the IGD group had significantly higher Y-IAT scores than the AUD and HC groups (*p* < 0.001).

Regarding neurocognitive domains, IQs in both the AUD and IGD groups were significantly lower than those in the HC group (*p* < 0.001). Because there were significant differences in IQ, BDI, and BAI scores—variables that were significantly associated with executive function—among the three groups, IQ, BDI, and BAI scores were adjusted in the analysis of group differences in executive functions. We found significant differences in TMT-B (*F* = 4.216, *p* = 0.018) and SWM strategy (*F* = 3.317, *p* = 0.041) scores among the groups. The IGD group had significantly higher TMT-B scores than HCs (*p* = 0.017), suggesting impaired cognitive shift ability in the IGD group. The AUD group had significantly higher SWM-strategy scores than those with IGD and HCs (*p* = 0.040), indicating impaired SWM ability in the AUD group.

### KYN Pathway Metabolites

**FIGURE 3:**
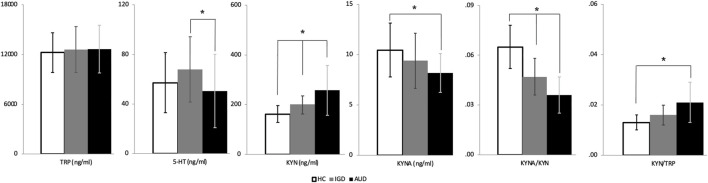
Kynurenine pathway metabolites in the alcohol use disorder, Internet gaming disorder, and healthy control groups. TRP, Tryptophan; 5-HT, 5-hydroxytryptamine; KYN, Kynurenine; KYNA, Kynurenic acid. *p<0.05.

Figure 3; Supplementary Table S3 show the serum levels of KYN pathway metabolites in each group. Because BDI and BAI scores were significantly associated with the serum levels of KYN pathway metabolites, the analysis of group differences in KYN pathway metabolites was adjusted for these scores. We found significant differences in 5-HT (*F* = 3.941, *p* = 0.023), KYN (*F* = 12.804, *p* < 0.001), KYNA (*F* = 3.639, *p* = 0.030), the KYNA/KYN ratio (*F* = 32.359, *p* < 0.001), and the KYN/TRP ratio (*F* = 12.516, *p* < 0.001) among the three groups. The AUD group showed significant increases in KYN levels and the KYN/TRP ratio (a measure of IDO activity indicating neurotoxicity) compared to HCs (*p* < 0.001 and *p* < 0.001, respectively). The IGD group exhibited levels of KYN intermediate between those of the AUD group and HCs (*p* < 0.001 and *p* = 0.020, respectively). Furthermore, the AUD group exhibited significant decreases in KYNA levels and the KYNA/KYN ratio (a measure of neuroprotective activity) compared to HCs (*p* = 0.033 and *p* < 0.001, respectively). The IGD group had a KYNA/KYN ratio intermediate between those of the AUD group and HCs (*p* = 0.001 and *p* < 0.001, respectively). Regarding 5-HT levels, the AUD group exhibited decreased serum 5-HT compared to the IGD group (*p* = 0.037). There was no significant difference in TRP among the groups.

### Relationships Among Clinical Variables, Executive Function, and KYN Pathway Metabolites

**FIGURE 4:**
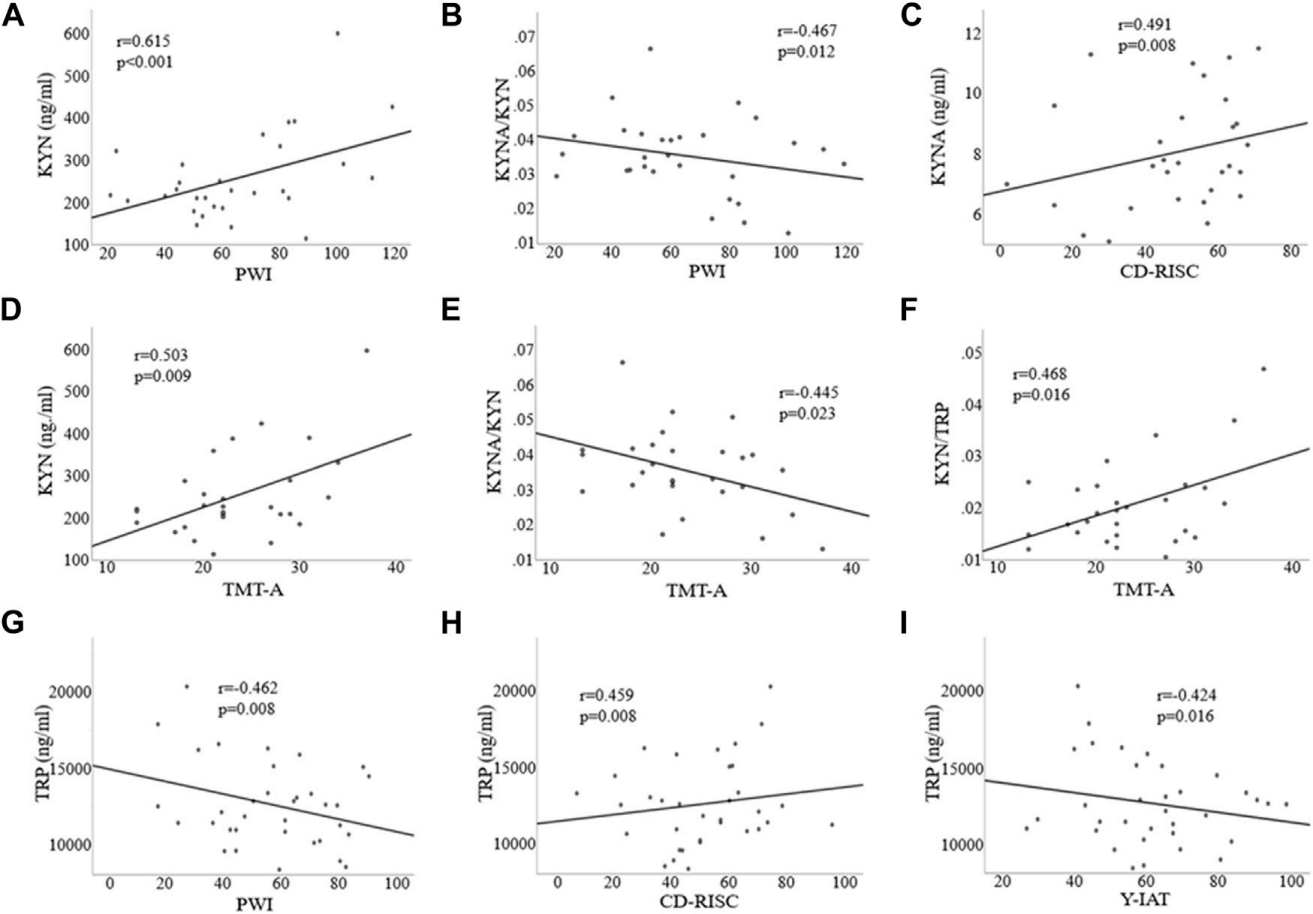
Correlations among clinical, executive function, and kynurenine pathway metabolite variables in the alcohol use disorder **(A–F)** and Internet gaming disorder **(G–I)** groups. TRP, Tryptophan; 5-HT, 5-hydroxytryptamine; KYN, Kynurenine; KYNA, Kynurenic acid; Y-IAT, Young’s Internet Addiction Test; PWI, Psychosocial Well-Being Index; CD-RISC, Connor-Davidson Resilience Scale; TMT-A, Trail Making Test-A.

Significant correlations between KYN and PWI (r = 0.615, *p* < 0.001; Figure 4A), KYNA and CD-RISC (*r* = 0.491, *p* = 0.008; Figure 4C), the KYNA/KYN ratio and PWI (*r* = –0.467, *p* = 0.012; Figure 4B) were found in patients with AUD. KYN/TRP ratio was also correlated with both AUDIT (*r* = 0.389, *p* = 0.041; data not shown in Figure, but please see Supplementary Figure S1B) and PWI (*r* = 0.731, *p* < 0.001; data not shown in Figure, but please see Supplementary Figure S1A) scores with BDI and BAI scores as covariates. In addition, significant correlations were found between KYN and TMT-A (*r* = 0.503, *p* = 0.009; Figure 4D), the KYNA/KYN ratio and TMT-A (*r* = –0.445, *p* = 0.023; Figure 4E), and the KYN/TRP ratio and TMT-A (r = 0.468, *p* = 0.016; Figure 4F) with the BDI and BAI scores and IQ as covariates in the AUD group.

In the IGD group, TRP was significantly correlated with Y-IAT (*r* = –0.424, *p* = 0.016; Figure 4I), PWI (*r* = −0.462, *p* = 0.008; Figure 4G), and CD-RISC (*r* = 0.459, *p* = 0.008; Figure 4H) scores. Furthermore, the KYN/TRP ratio was significantly correlated with PWI (r = 0.573, *p* = 0.001; data not shown in Figure, but please see Supplementary Figure S1A) and Y-IAT (r = 0.555, *p* = 0.001; data not shown in Figure, but please see Supplementary Figure S1C) scores, with BDI and BAI scores as covariates.

As an exploratory analysis, results of the simple linear regression analyses are provided in the supplementary materials.

### Mediation Analyses Among Stress Level, Resilience, and KYN Pathway Metabolites

In the AUD and HC groups, the simple mediation analysis of KYN with stress level as a mediator indicated that AUD had a total effect (*B* = 74.417, S.E = 27.564, *p* = 0.009) but no direct effect (*B* = 32.424, S.E = 25.674, *p* = 0.212). The effect of AUD on stress level (*B* = 15.501, S.E = 5.182, *p* = 0.004) and that of stress level on KYN were positively related (*B* = 2.709, S.E = 0.601, *p* < 0.001). The Sobel test indicated a significant indirect effect of stress level (*Z* = 2.493, *p* = 0.013). This significant indirect effect of 41.993 was confirmed by the bootstrap results with 5,000 resamples and a 95% bias-corrected and accelerated bootstrap interval (BCa CI) around this value, which did not contain zero (5.285, 101.078). The mediating effect of stress level on the relationship between AUD and KYN/TRP ratio had a significant total effect (*B* = 0.008, S.E = 0.002, *p* = 0.001) and direct effect (*B* = 0.005, S.E = 0.002, *p* = 0.028). There were positive effects of AUD on stress level (*B* = 15.501, S.E = 5.182, *p* = 0.004) and of stress level on the KYN/TRP ratio (B < 0.001, S.E < 0.000, *p* < 0.001). However, the Sobel test showed that the indirect effect of stress level was not significant (*Z* = 1.663, *p* = 0.096).

In the IGD and HC groups, the mediating effect of IGD on TRP with stress level as a mediator had no significant total effect (*B* = −369.480, S.E = 883.117, *p* = 0.677) or direct effect (*B* = 294.933, S.E = 895.759, *p* = 0.743). The effects of IGD on stress level (*B* = 13.283, S.E = 5.113, *p* = 0.012) and stress level on TRP (*B* = −50.020, S.E = 20.829, *p* = 0.019) were statistically significant. However, the Sobel test did not show a significant indirect effect of stress level (*Z* = −1.753, *p* = 0.078). The mediating effect of IGD on the KYN/TRP ratio with stress level as a mediator indicated a significant total effect (*B* = 0.004, S.E = 0.001, *p* = 0.002) and direct effect (*B* = 0.003, SE = 0.001, *p* = 0.027). The effects of IGD on stress level (*B* = 13.283, S.E = 5.113, *p* = 0.012) and stress level on the KYN/TRP ratio (*B* = 0.001, S.E < 0.001, *p* = 0.001) were statistically significant. The Sobel test showed that stress level had significant indirect effect (*Z* = 2.598, *p* = 0.009).

### Moderated Mediation Analyses Among Stress Level, Resilience, and KYN Pathway Metabolites

**FIGURE 5:**
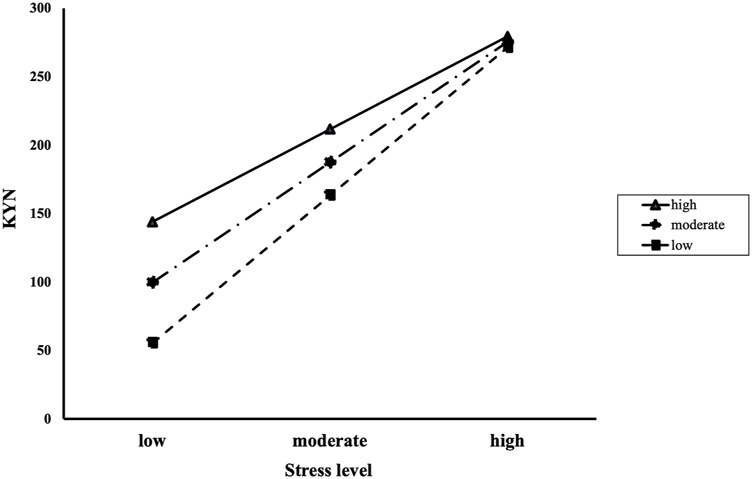
The interaction effects of stress level and resilience on KYN in the alcohol use disorder and healthy control groups. Participants with high resilience showed higher KYN with low (mean—1 S.D), moderate (mean), and high (mean +1 S.D) stress levels than those with moderate and low resilience. As stress level increased, the differences of KYN decreased according to resilience level. KYN: Kynurenine; S.D: standard deviation; Resilience and stress level were measured using the Connor-Davidson Resilience Scale and Psychosocial Well-Being Index, respectively.

We conducted a moderated mediation analysis with a significant mediation model, which was the association between AUD or IGD and KYN pathway metabolites with stress level as a mediator. Before conducting this analysis, we tested whether resilience had moderating effects on the relationship between stress level and KYN pathway metabolites. In the AUD and HC groups, the interaction effects of stress level and resilience on KYN were significant (*B* = −0.024, S.E = 0.012, *p* = 0.045). A significant conditional effect of stress level on KYN was found for low resilience (mean −1 S. D; *B* = 3.941, boot S.E = 0.706, *p* < 0.001), moderate resilience (mean; *B* = 3.465, boot S.E = 0.637, *p* < 0.001), and high resilience (mean +1 S. D; *B* = 2.990, boot S.E = 0.648, *p* < 0.001; Figure 5). In the IGD and HC groups, there was no significant the interaction effects of stress level and resilience on KYN/TRP ratio (B < 0.001, S.E < 0.001, *p* = 0.816).

The moderated mediation analysis revealed that resilience moderated the mediating effect of AUD on KYN through stress level [index of moderated mediation = −0.557, boot S.E = 0.331, BCa CI (−1.349, −0.081); Table 2]. The positive relationships between AUD and KYN via stress level were significant at low [mean −1 S. D; B = 58.567, boot S.E = 31.964, BCa CI (12.019, 133.806)] moderate [mean; B = 47.634, boot S.E = 27.552, BCa CI (8.383, 112.601)], and high [mean +1 S. D; B = 36.702, boot S.E = 24.099, BCa CI (3.796, 94.811)] levels of resilience. In other words, participants with AUD showed high-stress levels, which was related to an increase in KYN, whereas this tendency declined as resilience increased.

**TABLE 2:** Moderated mediation model with AUD, stress level and KYN pathway metabolites (*n* = 65, bootstrap = 5,000).

	B	S.E	*p*	95% CI	
				LLCI	ULCI
Mediator variable model (Y = stress level)					
AUD (predictor)	15.501**	5.182	0.004	5.131	25.871
Dependent variable model (Y = KYN)					
AUD (predictor)	64.591*	26.227	0.017	12.051	117.131
Stress level (Mediator)	3.073***	0.630	<0.001	1.810	4.336
Resilience (Moderator)	1.229	0.739	0.102	−0.252	2.710
Stress level × Resilience	-0.036**	0.012	0.005	−0.061	−0.011

	**B**	**Boot S.E**	**95% BCa CI**		
			**LLCI**	**ULCI**	
Conditional indirect association on stress level at different values of the resilience (moderator)					
Low (Mean—1 S.D)	3.778***	0.680	2.417	5.140	
Moderate (Mean)	3.073***	0.630	1.810	4.336	
High (Mean +1 S.D)	2.368**	0.670	1.025	3.711	
Moderated mediation effect	−0.557	0.331	−1.349	−0.081	

S.E, standard error; CI, confidence intervals; LLCI, lower limit confidence intervals; ULCI, upper limit confidence intervals; AUD, alcohol use disorder; 95% BCa CI, 95% bias-corrected and accelerated bootstrap interval; Resilience was measured by Connor-Davidson resilience scale (CD-RISC); stress level was meausered by Psychosocial Well-Being Index (PWI); Mean score and standard deviation of CD-RISC, were 62.616 and 19.504; BDI, BAI, IQ, were controlled.

*p* < 0.05*, *p* < 0.01**, *p* < 0.001***.

## Discussion

To the best of our knowledge, this is the first study to investigate psychoneuroimmunological characteristics in both substance and behavioral addictions. We demonstrated that both addictive disorder groups showed higher stress levels and lower resilience levels compared to the HC group. The AUD group had significantly higher scores for SWM strategy than the IGD group and HCs, indicating impaired SWM ability in the AUD group. The IGD group had significantly higher TMT-B scores than HCs, suggesting impaired cognitive shift performance in the IGD group. Importantly, the AUD group showed significant increases in KYN level and the KYN/TRP ratio as well as decreases in KYNA and the KYNA/KYN ratio compared to HCs. The IGD group exhibited KYN and KYNA/KYN ratio values intermediate between those of the AUD group and HCs. In patients with AUD, KYN metabolites were more extensively associated with stress levels and executive dysfunction than in the IGD group. Furthermore, the relationship between stress level and KYN increased with higher resilience in the AUD group.

Exposure to stress is an environmental factor that has been associated with the onset of addictive disorders (Koob and Schulkin, 2019; Fronk et al., 2020), and resilience is considered a protective factor against addictive disorders (Nam et al., 2018; Shin et al., 2019; Ersche et al., 2020). In the present study, both addictive disorder groups had higher levels of stress and lower resilience compared to HCs. These findings support the roles of stress and resilience in the pathophysiology of addictive disorders. In addition, stressors have been documented as affecting the immune system, and increases in various inflammatory markers have been reported for states of acute and chronic stress (O'Farrell and Harkin, 2017; Weik et al., 2008). These types of stress also increase the synthesis and secretion of adrenal cortisol hormones, which affect the KYN pathway in the peripheral system and brain, shifting TRP catabolism toward the neurotoxic metabolite QUIN (Savino et al., 2020). In the present study, the AUD group or IGD group exhibited increased serum levels of KYN and decreased KYNA/KYN ratios compared to HCs. KYN, a pivotal metabolite in the KYN pathway, is known to be metabolized via two distinct pathways: one is the neuroprotective KYNA branch via the enzyme KAT and the other is the neurotoxic branch leading to the production of QUIN via KMO (Stone, 1993). The presence of increased levels of KYN and decreased KYNA/KYN ratios in patients with both addictive disorders in the present study might reflect a metabolic shift toward neurotoxicity.

Interestingly, the AUD group exhibited much greater changes in KYN and the KYNA/KYN ratio than the IGD group. The AUD group also exhibited a significantly increased KYN/TRP ratio (a measure of IDO activity indicating stressful effects or neurotoxicity) and decreased levels of KYNA compared to HCs, while the IGD group exhibited levels intermediate between those of the AUD group and HCs. These findings suggest that stress-related psychoimmunological changes can be more severe in the AUD group than in those with IGD and HCs. Previous research revealed that KYN and the KYN/TRP ratio were higher in the AUD group than in the control group (Vidal et al., 2020). In the present study, the IGD group also exhibited increased levels of KYN, although they were not exposed to chronic alcohol use. Therefore, it appears that the metabolic shift toward neurotoxicity in patients with addictive disorders may be related to higher levels of chronic stress and lower resilience. On the other hand, chronic alcohol use leads to the activation of the immune system (de Timary et al., 2017; Pascual et al., 2015), and pro-inflammatory molecules activate IDO (Munn and Mellor, 2013). Greater changes in the AUD group relative to the IGD reflect the effects of chronic alcohol use on KYN pathway metabolites.

This study demonstrated the possible mediating role of stress and resilience on the effect of AUD on KYN levels. As shown in Figure 4, higher stress may impact KYN via indirect mechanisms. On the other hand, resilience plays a role in mitigating the effects of stress. Previous studies have reported associations between the KYN pathway and the progression of neurotoxicity. QUIN is one of the end products of the KYN pathway and is one of the most important metabolites in terms of biological activity and neurotoxicity. QUIN induces activation of NMDA receptors, increases neuronal activity and elevations of intracellular calcium concentration, and causes selective neuronal lesions in the hippocampus and striatum, which are brain regions closely associated with the memory and reward system (Schwarcz et al., 2012; Koob and Volkow, 2016). The results of this study revealed significant correlations between executive dysfunction and KYN pathway metabolites, including KYN, the KYN/TRP ratio, and the KYNA/KYN ratio in the AUD group. Zhou et al. (2019) reported that learning and processing speed in patients with depression were associated with KYN serum levels and the KYN/TRP ratio, potentially implicating the KYN pathway in the pathological mechanism of cognitive dysfunction. We also found that resilience was positively correlated with KYNA levels. KYNA, as an NMDA and a7nACh receptor antagonist, is associated with cognitive function. The association of KYNA with cognition in depressive disorders may be linked to alterations in a7nACh receptor activity (Anderson, 2018). Therefore, higher stress levels and low resilience levels could aggravate the progression of neurotoxicity through changes in KYN pathway metabolites. This distortion may be linked to neurocognitive dysfunction in AUD.

In the case of IGD, significant correlations were found between serum TRP levels and stress levels, resilience, and symptom severity. TRP metabolism is considered the “heart of the whole complex network between the immune and neuroendocrine systems” because it plays an important role in many pathophysiological processes such as neurotransmission, inflammation, oxidative stress, and the immune response (Myint and Kim, 2014). Higher stress and lower resilience can influence the depletion of TRP, which could lead to excessive gaming in patients with IGD, although this interpretation should be confirmed by longitudinal studies in the future.

The present study has several limitations. First, this study was a cross-sectional study, so causal relationships between stress, resilience, executive function, symptom severity, and KYP pathway metabolites should be confirmed in further studies. Additionally, the symptom severity, levels of stress and resilience were obtained through self-report questionnaires. Second, KYN metabolites were detected in the peripheral blood rather, not than in the cerebrospinal fluid. Changes in peripheral KYN pathway functioning are not considered identical to changes in central KYN pathway functioning. Third, depressive mood, anxiety and obesity may influence the KYN pathway in patients with addictive disorders. To exclude this influence, we controlled for the effect of mood on KYN pathway metabolites in the analysis. However, body weights of participants were not measured. Finally, we could not analyze QUIN serum levels as a neurotoxic marker. Instead, we considered KYN and the KYN/TRP ratio to be neurotoxic metabolites. Future research should validate the effects of stress and resilience on KYN pathway metabolites, including QUIN.

The present findings indicate that stress may induce an imbalance in downstream KYN pathway metabolites and neurotoxic changes that create a vulnerable neuronal network, which may contribute to the development of addictive disorders such as AUD and IGD. The AUD group exhibited more changes in KYN pathway metabolites than the IGD group, which suggests chronic alcohol use influences the relationship between stress and KYN pathway metabolites in AUD. Among the KYN pathway metabolites, the KYN/TRP ratio can be a neuromediator between stress and behavioral changes in both addictive disorders. Regulation of the KYN pathway may be of critical importance and may candidate an important target for treatment modalities for addictive disorders.

## Data Availability

The raw data supporting the conclusion of this article will be made available by the authors, without undue reservation.
