# Supplementary material for: The Kynurenine Pathway and Mediating Role of Stress in Addictive Disorders: A Focus on Alcohol Use Disorder and Internet Gaming Disorder
Source: Front Pharmacol. 2022 Apr 11;13:865576. doi: 10.3389/fphar.2022.865576 (PMC9037037; doi:10.3389/fphar.2022.865576)
Supplement: Supplementary file 1 [file DataSheet1.docx]

**Supplementary Materials**

**Results**

**Relationships among clinical variables, executive function, and KYN pathway metabolites**

Significant results of the simple linear regression analyses among variables revealed that PWI score was predictive of KYN (*β =* 0.442, p = 0.014) and the KYN/TRP ratio (*β =* 0.416, p = 0.022) in the AUD group. Furthermore, the KYN/TRP ratio was predictive of TMT-A score (*β =* 0.392, p = 0.036) and the severity of addiction symptoms as measured by AUDIT (*β =* 0.403, p = 0.027) in the AUD group. In addition, KYN (*β =* 0.449, p = 0.015) and KYNA (*β =* −0.402, p = 0.031) were predictive of TMT-A score. In the IGD group, PWI score was predictive of the KYN/TRP ratio (*β =* 0.375, p = 0.029). Furthermore, the KYN/TRP ratio was predictive of the severity of addiction symptoms as measured by the Y-IAT score (*β =* 0.384, p = 0.025) (supplementary Figure 1).

No significant relationships among the other variables were found in patients with AUD or IGD.

**Supplementary Table 1. Multiple reaction ion monitoring (MRM) transition parameters for the analytes and their internal standards**

| Analyte | RT (min) | Q1 (***m/z*)** | Q3 (***m/z*)** | DP^1)^ (V) | CE^2)^ (V) | CXP^3)^ (V) |
| --- | --- | --- | --- | --- | --- | --- |
| TRP | 3.51 | 205.0 | 188.1, 146.0 | 31 | 13, 21 | 10, 12 |
| 5-HT | 3.04 | 177.1 | 160.1, 115.0 | 16 | 13, 37 | 8, 14 |
| KYN | 3.1 | 209.1 | 192.1, 146.1 | 31 | 13, 27 | 12, 18 |
| KYNA | 3.65 | 190.0 | 144.0, 115.9 | 116 | 25, 43 | 20, 54 |
| TRP-^13^C_11_ | 3.51 | 216.1 | 199.1 | 1 | 13 | 12 |
| 5-HT-d_4_ | 3.03 | 181.0 | 164.1, 136.1 | 1 | 11, 31 | 8, 8 |
| KYNA-d_5_ | 3.65 | 194.9 | 149.1, 177.1 | 1 | 27, 17 | 14, 10 |

^1)^ Declustering potential, ^2)^ Collision energy, ^3)^ Cell exit potential. Ion pairs for MRM and different parameters including declustering potentials, entrance potentials, collision energies, and cell exit potentials for each analyte were investigated for better response. TRP: tryptophan; 5-HT: 5-hydroxytryptamine; KYN: kynurenine; KYNA: kynurenic acid

**Supplementary Table 2. Calibration parameters for the measurement of analytes**

| Analyte | Used internal standard | Linearity range  (ng/mL) | Standard curve linearity (r^2^) | LOD  (ng/mL) | LOQ  (ng/mL) |
| --- | --- | --- | --- | --- | --- |
| TRP | TRP-^13^C_11_ | 2.44-156.25 | 0.9996 | 0.74 | 2.44 |
| 5-HT | 5-HT-d_4_ | 1.95-125 | 0.9999 | 0.59 | 1.95 |
| KYN | TRP-^13^C_11_ | 1.95-125 | 0.999 | 0.59 | 1.95 |
| KYNA | KYNA-d_5_ | 0.49-31.25 | 0.9995 | 0.15 | 0.49 |

*The limit of detection (LOD), defined as the concentration level with the signal-to-noise ratio at 3, and the limit of quantification (LOQ), defined as the concentration level with the signal-to-noise ratio at 10, were determined from calibration curves. TRP: tryptophan; 5-HT: 5-hydroxytryptamine; KYN: kynurenine; KYNA: kynurenic acid

**Supplementary Table 3. Kynurenine pathway metabolites in the alcohol use disorder, Internet gaming disorder and healthy control groups**

|  | Healthy control | Internet gaming disorder | Alcohol use disorder |  |  |  |
| --- | --- | --- | --- | --- | --- | --- |
|  | N=35 | N=34 | N=30 | F | P | Post-hoc |
|  | mean ± SD | mean ± SD | mean ± SD |  |  |  |
| TRP (ng/mL) | 12252.368 ± 2398.982 | 12610.111 ± 2790.534 | 12666.295 ± 2897.017 | 0.031 | 0.970 |  |
| 5-HT (ng/mL) | 57.180 ± 24.276 | 68.005 ± 26.376 | 50.442 ± 29.517 | 3.941 | 0.023* | A<I |
| KYN (ng/mL) | 161.266 ± 33.809 | 198.530 ± 35.749 | 257.594 ± 100.290 | 12.804 | <0.001* | A>I>H |
| KYNA (ng/mL) | 10.448 ± 2.661 | 9.379 ± 2.725 | 8.179 ± 1.941 | 3.639 | 0.030* | A<H |
| KYNA/KYN ratio | 0.065 ± 0.013 | 0.047 ± 0.011 | 0.036 ± 0.011 | 32.359 | <0.001* | A<I<H |
| KYN/TRP ratio | 0.013 ± 0.003 | 0.016 ± 0.004 | 0.021 ± 0.008 | 12.516 | <0.001* | A>H |

TRP: tryptophan; 5-HT: 5-hydroxytryptamine; KYN: kynurenine; KYNA: kynurenic acid

P-value was adjusted by BDI and BAI. The Bonferroni test was used for post hoc analyses.

*p<0.05


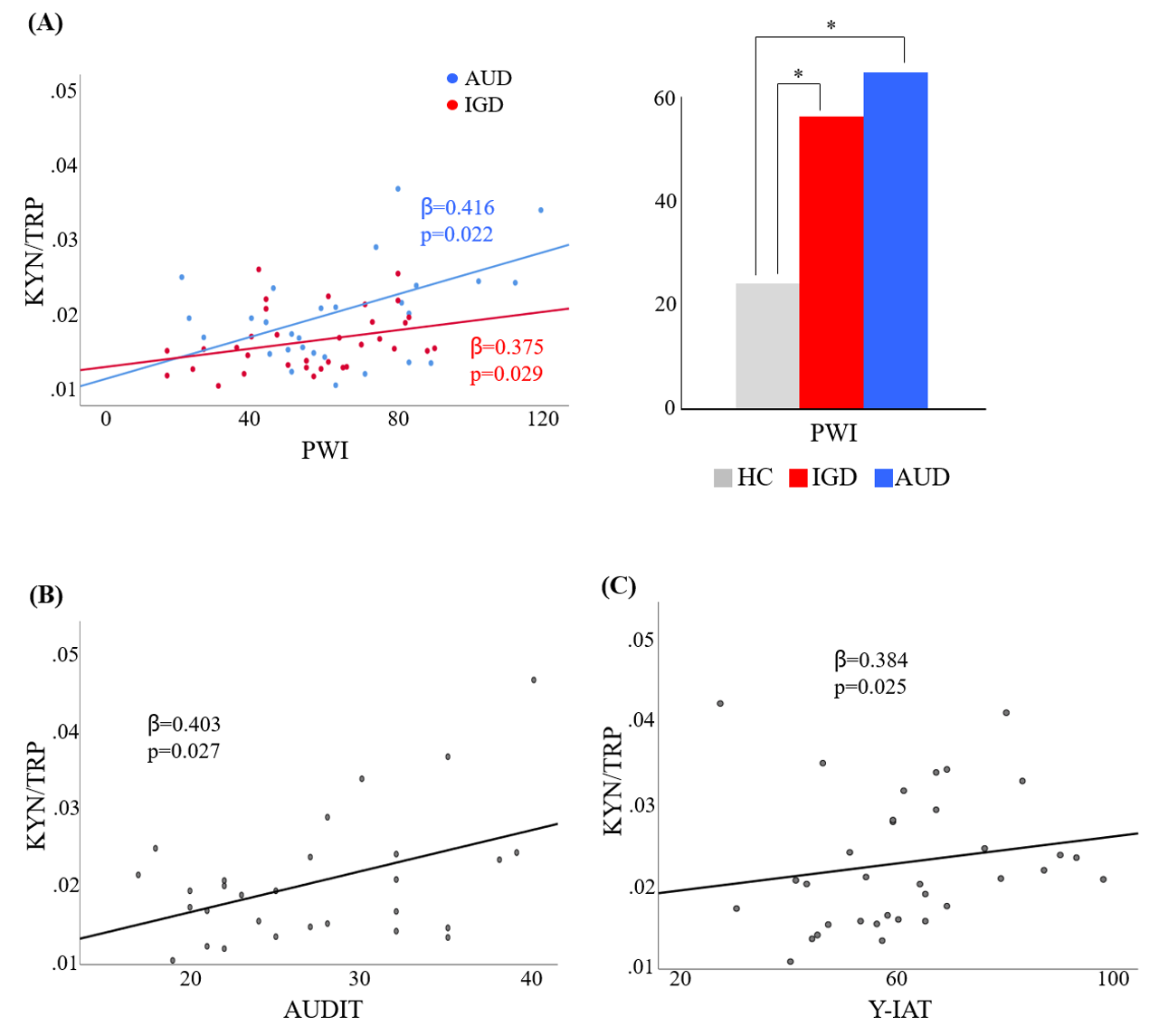


**Supplementary Figure 1. Relationships among level of stress, kynurenine/tryptophan ratio and severity of addiction symptom in the alcohol use disorder and Internet gaming disorder groups. (A) Associations between level of stress and kynurenine/tryptophan ratio in each group. (B) Associations between kynurenine/tryptophan ratio and severity of addiction symptom measured by AUDIT in the alcohol use disorder group. (C) Associations between kynurenine/tryptophan ratio and severity of addiction symptom measured by Y-IAT in the Internet gaming disorder group**

AUD: Alcohol Use Disorder; IGD: Internet Gaming Disorder; HC: Healthy Control; PWI: Psychosocial Well-being Index; KYN: Kynurenine; TRP: Tryptophan; AUDIT: Alcohol Use Disorder Identification Test; Y-IAT: Young’s Internet Addiction Test

*p<0.05
